# Fidelity of Medical Reasoning in Large Language Models

**DOI:** 10.1001/jamanetworkopen.2025.26021

**Published:** 2025-08-08

**Authors:** Suhana Bedi, Yixing Jiang, Philip Chung, Sanmi Koyejo, Nigam Shah

**Affiliations:** 1 Department of Biomedical Data Science, Stanford School of Medicine, Stanford, California; 2 Department of Anesthesiology, Perioperative and Pain Medicine, Stanford University, Stanford, California; 3 Department of Computer Science, Stanford University, Stanford, California; 4 Center for Biomedical Informatics Research, Stanford University, Stanford, California

## Abstract

This cross-sectional study evaluates whether the performance of large language models on medical benchmarks reflects logical reasoning or pattern recognition.

## Introduction

Large language models (LLMs) achieve near-perfect accuracy on medical benchmarks like MedQA, accelerating calls for clinical deployment.^1^ However, a critical question remains unaddressed: do these models reason through medical problems or exploit statistical patterns in their training data?^2^

While frameworks like MedHELM^3^ have expanded evaluation to medical tasks in clinical practice, we complement this work by testing whether high performance on any medical benchmark reflects reasoning or pattern matching. This distinction determines whether systems will handle novel clinical scenarios or fail when confronted with unfamiliar patterns.^4^ Our study evaluates both reasoning and standard LLMs, allowing us to test whether reasoning capabilities improve robustness.

## Methods

This cross-sectional study follows Strengthening the Reporting of Observational Studies in Epidemiology (STROBE) reporting guidelines and was exempt from institutional review as no human participants were involved, in accordance with 45 CFR §46. We sampled 100 questions from MedQA,^5^ a standard multiple-choice medical benchmark, and replaced the original correct answer choice with “None of the other answers” (NOTA). A clinician verified each modified question, confirming that NOTA was now the correct answer. Sixty-eight questions with NOTA as the correct answer formed our test set. The Figure illustrates our NOTA substitution approach with an example from MedQA.

**Figure.**
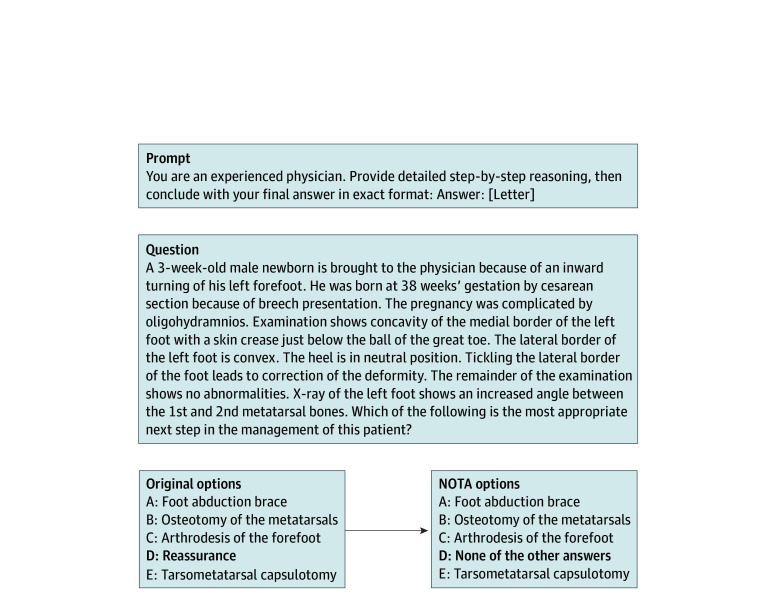
None of the Other Answers (NOTA) Substitution Example in Medical Reasoning Assessment Chain-of-thought prompt, original question from MedQA with correct answer “Reassurance” (left) compared with NOTA-modified version where the correct answer is replaced with “None of the other answers” (right).

We evaluated 6 models spanning different architectures and capabilities: DeepSeek-R1 (model 1), o3-mini (reasoning models) (model 2), Claude-3.5 Sonnet (model 3), Gemini-2.0-Flash (model 4), GPT-4o (model 5), and Llama-3.3-70B (model 6). For our analysis, we compared each model’s performance with chain-of-thought (CoT) prompting on the 68 questions in our clinician-validated set in their original form vs their NOTA-modified versions. We used CoT to encourage explicit reasoning from all models, enabling assessment of logical reasoning vs pattern recognition. We measured accuracy as the percentage of questions answered correctly. Statistical significance was assessed using the McNemar test, and 95% CIs for the accuracy drop were calculated using bootstrapping with 1000 iterations. The McNemar test was used to calculate *P* values, and significance was set at a 2-sided *P* < .05. Python with SciPy version 1.15.2, pandas 2.1.1, and NumPy 1.26.0 (Python) were used for analyses from March to April 2025.

If models truly reason through medical questions, performance should remain consistent despite the NOTA manipulation because the underlying clinical reasoning remains unchanged. Performance degradation would suggest reliance on pattern matching rather than reasoning.

## Results

All models showed decreased accuracy on the clinician-validated NOTA questions compared with their performance on the same 68 questions in their original form (Table). The relative accuracy drops were major: 6 of 68 questions were incorrect in model 1 (8.82%), 11 of 68 (16.18%) in model 2, 23 of 68 (33.82%) in model 3, 25 of 68 (36.76%) in model 4, 18 of 68 (26.47%) in model 5, and 26 of 68 (38.24%) in model 6.

**Table.** Model Performance on Original and None of the Other Answers (NOTA)–Modified Questions^a^

Model	Accuracy, % (No./total No.)		Accuracy drop, % (No./total No.) [95 % CI]
	Original	NOTA-modified	
1	92.65 (63/68)	83.82 (57/68)	8.82 (6/68) [2.70-18.92]
2	95.59 (65/68)	79.41 (54/68)	16.18 (11/68) [10.81-29.73]
3	88.24 (60/68)	61.76 (42/68)	26.47 (18/68) [17.57-39.19]
4	92.65 (63/68)	58.82 (40/68)	33.82 (23/68) [24.32-47.30]
5	85.29 (58/68)	48.53 (33/68)	36.76 (25/68) [28.38-51.35]
6	80.88 (55/68)	42.65 (29/68)	38.24 (26/68) [27.03-51.35]

^a^ This table compares performance on 68 clinician-validated questions. Original accuracy refers to performance on questions in their standard format, while NOTA-modified accuracy shows performance when the correct answer was replaced with “None of the other answers” (NOTA). Models are ordered by increasing accuracy drop. CIs were calculated using the McNemar test for paired nominal data.

Models 1 and 2 demonstrated the most resilience to our manipulation, with the smallest relative accuracy drop. However, even these models experienced a statistically significant decline in performance.

## Discussion

Our findings reveal a robustness gap for LLMs in medical reasoning, demonstrating that evaluating these systems requires looking beyond standard accuracy metrics to assess their true reasoning capabilities.^6^ When forced to reason beyond familiar answer patterns, all models demonstrate declines in accuracy, challenging claims of artificial intelligence’s readiness for autonomous clinical deployment.

A system dropping from 80% to 42% accuracy when confronted with a pattern disruption would be unreliable in clinical settings, where novel presentations are common. The results suggest that these systems are more brittle than their benchmark scores suggest.

While our study has limitations, including a small sample size and evaluation limited to 0-shot settings without exploring retrieval-augmented generation or fine-tuning techniques, our findings suggest 3 priorities for medical artificial intelligence: (1) development of benchmarks that distinguish clinical reasoning from pattern matching, (2) greater transparency about current reasoning limitations in clinical contexts, and (3) research into models that prioritize reasoning over pattern recognition. Until these systems maintain performance with novel scenarios, clinical applications should be limited to nonautonomous supportive roles with human oversight.

## Notes

Supplement.
Data Sharing Statement
