# Supplementary material for: Fidelity of Medical Reasoning in Large Language Models
Source: JAMA Netw Open. 2025 Aug 8;8(8):e2526021. doi: 10.1001/jamanetworkopen.2025.26021 (PMC12334947; doi:10.1001/jamanetworkopen.2025.26021)
Supplement: Supplement. — Data Sharing Statement [file jamanetwopen-e2526021-s001.pdf]

Data Sharing Statement

Bedi. Fidelity of Medical Reasoning in Large Language Models. *JAMA Netw Open*. Published August 08, 2025. doi:10.1001/jamanetworkopen.2025.26021

Data
Data available: Yes
Data types: Data (not involving human participants)
How to access data: https://github.com/som-shahlab/med-nota
When available: With publication

Supporting Documents
Document types: Statistical/analytic code
How to access documents: https://github.com/som-shahlab/med-nota
When available: With publication

Additional Information
Who can access the data: anyone requesting the data
Types of analyses: for any purpose
Mechanisms of data availability: without investigator support
